# The Thermal Structural Transition of α-Crystallin Inhibits the Heat Induced Self-Aggregation

**DOI:** 10.1371/journal.pone.0018906

**Published:** 2011-05-09

**Authors:** Giuseppe Maulucci, Massimiliano Papi, Giuseppe Arcovito, Marco De Spirito

**Affiliations:** Istituto di Fisica, Universitá Cattolica S. Cuore, Roma, Italy; Dalhousie University, Canada

## Abstract

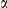
-crystallin, the major constituent of human lens, is a member of the heat-shock proteins family and it is known to have a quaternary structural transition at 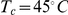. The presence of calcium ions and/or temperature changes induce supramolecular self-aggregation, a process of relevance in the cataractogenesis. Here we investigate the potential effect of the bovine 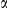-crystallin's structural transition on the self-aggregation process. Along all the temperatures investigated, aggregation proceeds by forming intermediate molecular assemblies that successively aggregate in clusters. The final morphology of the aggregates, above and below 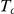, is similar, but the aggregation kinetics are completely different. The size of the intermediate molecular assemblies, and their repulsive energy barrier show a marked increase while crossing 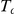. Our results highlight the key role of heat modified form of 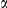-crystallin in protecting from aggregation and preserving the transparency of the lens under hyperthermic conditions.

## Introduction

Cataract, the opacity of the eye lens, is an age-onset pathology that affects nearly 50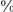 of the world's population over the age of 65, and is the leading cause of blindness worldwide [1]. Pathological studies of cataractous lenses have revealed that cataracts are composed of protein aggregates that precipitate in eye lens cells. The prevalent proteins within the eye lens are the crystallins. Lens transparency is thought to be maintained by a liquid-like, short range order present in highly concentrated solutions of these proteins [2], [3]. In mammals, there are three classes of crystallins denoted 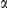, 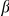, and 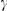 of which 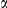-crystallin is the most abundant. 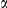-crystallin is an oligomeric protein, having a molecular mass of about 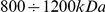, composed of two types of subunits, 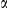
A and 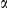
B crystallins, each having a molecular mass of about 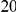 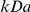 and 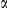
A crystallins in a ratio of 3 to 1 with respect to 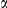
B [4]. The increase in light scattering in old and cataractous lenses can be ascribed to alterations in lens crystallins interactions due to age related post-translational modification of 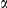-crystallin [5]–[8]. The alterations are triggered by lens cells exposition to elevated temperatures or other stress factors like 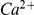 ions, that disrupt the liquid-like molecular order and promote the formation of large scattering particles [9], [10], following pathways that include both changes in the secondary structure and in the state of assembly [11], [12].

To monitor the heat and 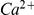 induced changes that occur in the structural domain of lens-crystallin different techniques like circular dichroism, fluorescence, Small Angle X-Ray scattering (SAXS) were used [13], [14]. Differential scanning calorimetric studies on 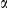-crystallin [14] show two endothermic transitions, a first ranging from 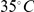 to 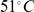, peaked at 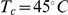 and a second major transition peaked at 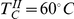. The transition at 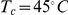 has been found to be biologically relevant [15]. At this temperature, 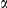-crystallin undergoes a minor change in its tertiary structure accompanying the exposure of its hydrophobic surfaces [16], [17], whereas its secondary structure is relatively unchanged.

Here we focused on the effects of this structural transition on the 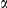-crystallin self-aggregation. Several aggregations have been induced by changing temperature, and therefore, by generating different heat-modified 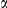-crystallin forms [18]. At temperature larger than 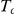 the kinetic pattern of the 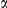-crystallin aggregation and the structural features of the clusters can be described according to the reaction limited cluster-cluster aggregation theory (RLCA) [19]. Aggregation occurs by initially forming the basic aggregation units, the high molecular weight forms of 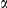-crystallin (HMW) [20], that successively continue to diffuse, collide and form rather compact fractal aggregates (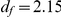). Although the final morphology of the aggregates is similar, the aggregation kinetics are completely different below and above 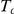, together with the size of the HMW, and their repulsive energy barrier (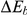). An abrupt increase in (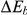) reveals a mechanism that markedly protects from aggregation preserving the transparency of the lens.

## Materials and Methods

### Preparation and aggregation of 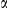-crystallin


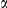
-crystallin from bovine eye lens was prepared according to Andreasi et al. [19].

The 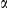-crystallin fractions suspended in 10 mM Tris-HCl buffer, pH 7.4, were thoroughly mixed and pooled together. The purified protein was divided into aliquots and kept in the same buffer at 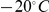 until used. Just before the experiment, the samples were thawed and centrifuged at 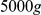
(Eppendorf 5418) for 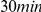 at 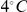, and the supramolecular aggregates already formed were discarded. The supernatant was filtered through a 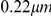 Millipore low-retention filter directly into the measuring cuvette. Protein concentration was determined by using an absorption coefficient of 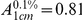 at 280 nm [21]. Aggregation of 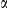-crystallin (1.0 mg/ml) was induced by quenching samples at the desiderated temperature and by the addition of 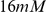 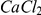. Indeed heating provokes the generation of particularly reactive isoforms of 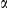-crystallin [14], and calcium ions stabilize the aggregates while they are forming and allow their continuous growth [9]. The whole set of measurements have been performed on different aliquots of the same sample. Five aggregations process for each temperature have been followed.

### Dynamic light scattering

Dynamic light scattering (DLS) (24) provides information on the aggregation kinetics and on the clusters dimension and evolution as the aggregation proceeds. DLS measurements were performed during aggregation by using a commercial computer-interfaced scattering instruments ALV/SLS-5000 system from ALV, Langen, Germany, equipped with a 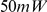 HeNe laser operating at 632.8 nm. The beam was focused to a spot (at 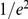) of 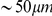 and the detection of the scattered light was carried out with a mono-mode fiber coupled to a photomultiplier, both mounted on a stepping motor-controlled rotating arm. The sample was contained in a cylindrical cell (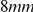 inner diameter) immersed in a toluene-filled index-matching vat whose temperature was controlled with a resolution of 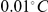. In this instrument, the usable wavevector range varies from 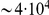 to 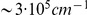. The autocorrelation function of the photopulses were performed by a 256-channel digital correlator (ALV-5000). Counts per second were used to measure the scattered intensity during the aggregation. DLS technique measures the intensity autocorrelation function 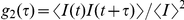 where 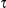 is the lag time and brackets represent the ensemble average [22]. The 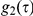 can be related to the field autocorrelation function 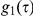 through the Siegert relation 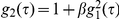 where 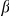 is an instrumental constant (in our set-up 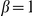). The mathematical form of 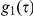 depends on the physical properties of the investigated system. For monodisperse particles, the electric field autocorrelation function decays exponentially following 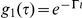,where the decay rate 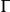 depends on the particle translational diffusion coefficient according to 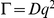. For a polydisperse sample, 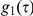 is more complex than a single exponential. In this condition, the derivative of 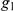 measures the intensity weighted average decay rate of the clusters:
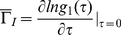
(1)


To determine 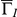 experimentally, we fitted the logarithm of the measured autocorrelation function 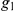, to a second-order polynomial, according to the cumulant expansion [23]:
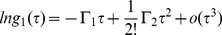
(2)


Where we assumed 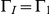. In aggregating systems, because of cluster-mass polydispersity, what we actually measure is an average effective diffusion coefficient that can be expressed as:
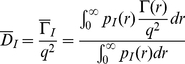
(3)where 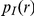 is the normalized intensity-weighted radius distribution function describing the distribution of the fraction of the intensity scattered by a particle of hydrodynamic radius 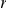 and decay rate 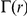, given by:
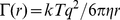
(4)where 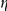 is the water viscosity and 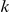 the Boltzmann constant. The intensity weighted average effective hydrodynamic radius 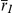 can be obtained using Stokes-Einstein Relation
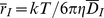
(5)


The complete distribution of decay rates can also be recovered by introducing from the relation [22], [24]:
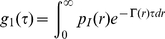
(6)


The recovery of the 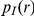 distribution, a classical ill posed problem, can be obtained by performing a regularized Laplace inversion of the intensity autocorrelation function using the software CONTIN [25]. In the presence of highly polydisperse fractal clusters, we also have to account for the volume and for the inner structure of clusters, therefore we need a complete recovery of the normalized number-weighted radius distribution function 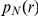 [24]:
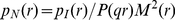
(7)where 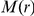 is the mass of a cluster of hydrodynamic radius 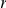 and 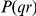 is the scattering form factor of the particle. Finally once 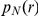 is known, the mean hydrodynamic radius can be easily determined by:
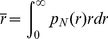
(8)


### Reaction limited cluster aggregation

A key to the understanding of proteins aggregation is the behavior of the energy of interaction between two approaching particles. It has been demonstrated that for a wide variety of proteins, this can be understood within the Derjaguin-Landau-Verwey-Overbeek (DLVO) model [26]. The key parameter is the repulsive energy barrier between two approaching particles. If the height of this energy barrier 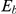 is sufficiently large compared to 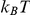, the particles will be unable to stick to one another when their diffusive motion causes them to collide, and the particle will be stable against aggregation. If 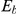 is reduced to much less than 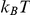, every collision will result in the particles sticking together, leading to very rapid aggregation, limited only by the rate of diffusion induced collisions between the clusters. This regime is therefore called diffusion-limited colloid aggregation (DLCA) [27]–[29]. For DLCA, computer simulation and several different experimental techniques show that clusters, characterized by a typical fractal dimension 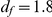, are essentially monodisperse in that their mass distribution is bell shaped and peaked around an average mass value, which grows linearly with time. By contrast, if 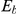 remains comparable to, or larger than, 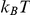, many collisions must occur before two particles can stick to one another. In this case, the aggregation rate is limited by the probability of overcoming the repulsive barrier 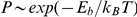 (sticking probability), leading to much slower aggregation. In this regime, called reaction limited aggregation (RLCA) [30]–[32], clusters have a structure more dense than in the DLCA, and with the typical 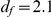. In each case, however, as particles stick together to become clusters, the clusters themselves continue to diffuse, collide, and aggregate. Each of these two regimes is characterized by a different time evolution of the average cluster mass, 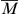, of the shape of cluster-mass distribution function, and of the fractal dimension of the resulting clusters. In RLCA regime the average cluster radius is an exponential function of time [19], [30], [33]:
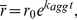
(9)where 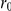 is the basic aggregation units and the aggregation rate 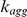 is a constant that depends on the sticking probability and therefore on the repulsive energy barrier [30], [34]:
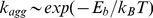
(10)


Clusters formed in the RLCA regime show an extremely high mass polydispersity, described by a power law, up to a cutoff mass 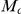, after which it decreases exponentially following:
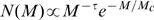
(11)where 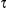 is a characteristic exponent (i.e 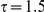 in RLCA) [30]–[32]. The DLCA and RLCA regimes must be considered universal in that their features do not depend on the nature of interacting forces between particles. Spectroscopic and microscopy techniques usually detect the normalized number-weighted radius distribution function, 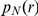, instead of the cluster-mass distribution function, 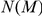. For a direct comparison of the theoretical predictions with experimental results, we here developed the relation between these two distributions. To this end let us to recall that by definition, 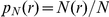, where 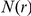 is the number of clusters of radius 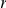 and 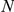 is the total number of clusters (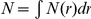). Since 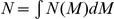, for fractal clusters we obtain 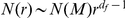. In the case of RLCA aggregation we obtain:
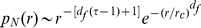
(12)where 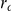 is the cut-off radius of clusters with a mass 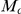.

## Results

**Figure 1:**
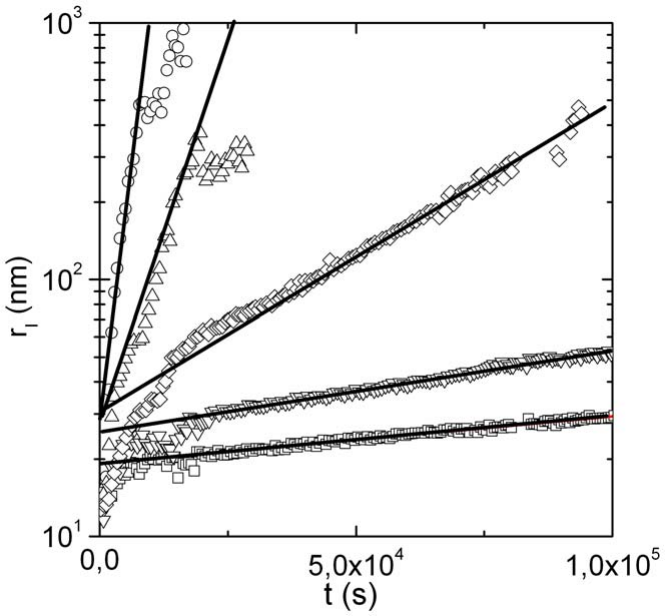
Time evolution of the average hydrodynamic radius of the clusters, 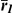, determined according to Eq.8 and Eq.12. Results for samples at 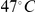 (squares), 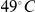
(inverted triangles), 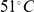 (diamonds), 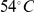 (triangles), 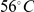 (circles), all above the characteristic temperature 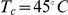 are reported. Solid lines are Fit of Eq.9 to experimental data.

To characterize the extent of the aggregation process, we performed dynamic light scattering experiments by measuring the time evolution of the intensity weighted average hydrodynamic radius of the clusters, 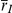, determined according to Eq.2, Eq.3, and Eq.5. The results for samples at different temperatures above 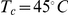 are reported in Fig. 1.

After an initial, fast, increase of 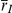 a second, slower, exponential growth, is observed.

The first increase of 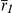 is ascribed to the initial conversion of the protein from the native to the heat- and calcium-induced conformers that rapidly bind to form high molecular weight species (HMW) [19]. The second exponential growth, already investigated in detail [19], is consistent with an RLCA process where HMWs, of radius 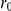, after a large number of collisions can stick together. The time at which appears the crossing between these two steps it is called 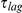.

Fits of Eq.9 to experimental data allow to recover 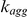, 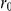 for each aggregation process (Table 1). The value of 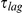 is determined as the 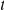 value where 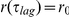.

**Table 1:** 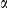
-crystallin aggregation constants.

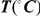	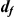	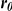 (nm)	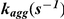	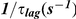
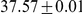	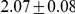	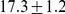	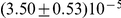	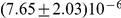
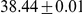	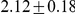	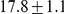	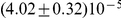	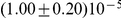
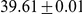	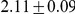	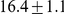	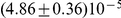	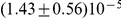
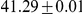	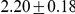	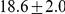	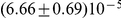	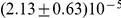
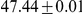	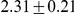	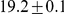	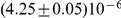	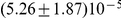
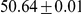	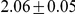	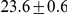	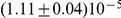	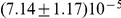
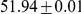	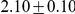	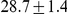	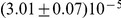	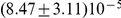
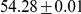	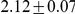	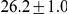	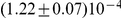	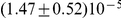
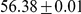	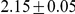	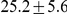	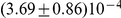	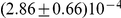

All the aggregations, carried out at different temperatures, show the same behavior. The initially formed basic aggregation units aggregate forming fractal clusters, accordingly to an RLCA process characterized by a temperature dependent rate constant (i.e. higher is the temperature faster is the aggregation rate). The size of basic aggregation units,instead, is independent on temperature with an average value of 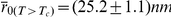.

**Figure 2:**
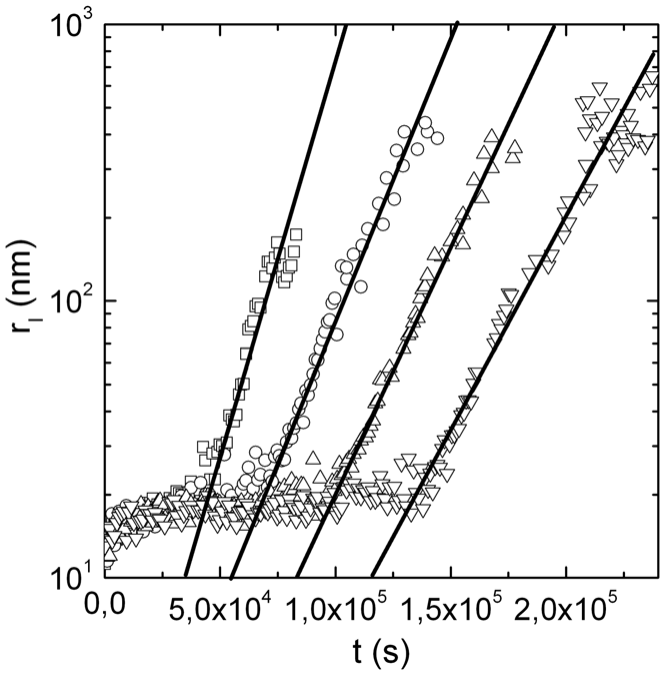
Time evolution of the average hydrodynamic radius of the clusters, 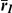, determined according to Eq.8 and Eq.12. Results for samples at 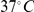 (inverted triangles), 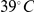
(triangles), 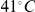 (circles), 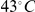 (squares), all below the characteristic temperature 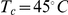 are reported. Solid lines are Fit of Eq. 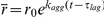 to experimental data.

**Figure 3:**
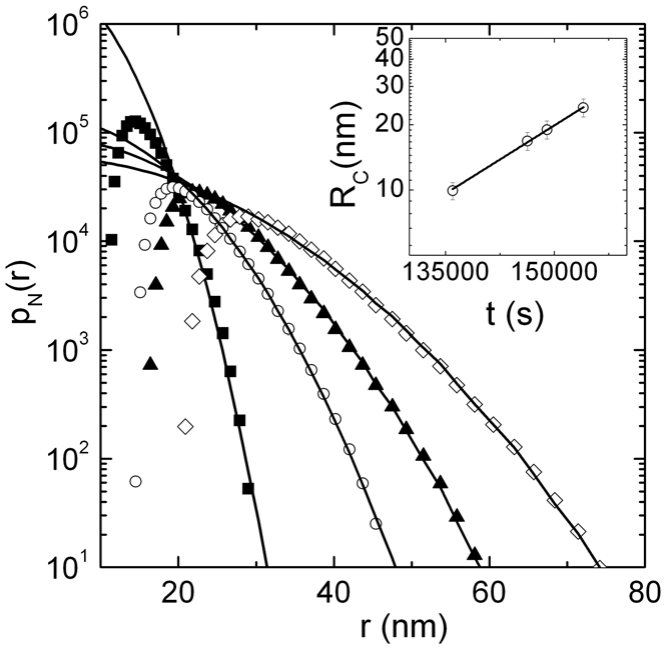
Representative distributions of 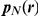 taken at different times (t = 136000 s black squares, t = 146300s open circles, t = 149000 s black triangles, t = 154000 s open diamonds) during the aggregation occurred at 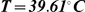. Fit to the Experimental data (solid lines) according the equation 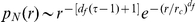 are reported. 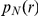 depends only on the cut-off radius 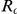 that increases exponentially with time according to the relation 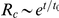 (inset), while is independent from the detailed nature of the aggregating particle as confirmed by the fractal dimension that does not vary as aggregation proceeds, keeping an average value of 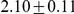.

**Figure 4:**
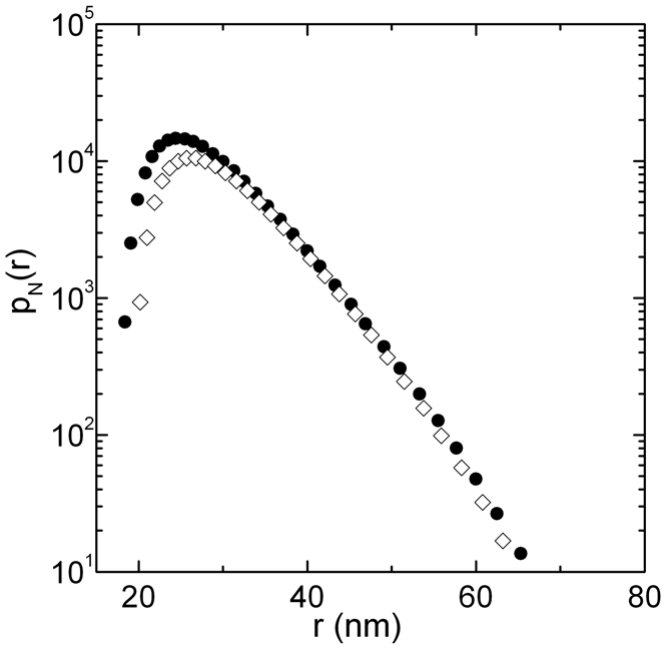
Aggregate's size distributions, recovered from aggregations occurred at T = 37C (full dots) and at T = 51C (open diamonds) below and above 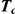, when the average radius reach a specific value 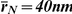. The overlap of the aggregates size distribution curves before and after the structural transition is an evidence that the shape of size distribution of aggregates depends only on the cluster-cluster interaction potential (i.e DLVO in our case) while temperature simply modulate the extent of aggregation.

By decreasing temperature below 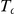 the time evolution of the aggregation process undergoes to a dramatic modification (Fig. 2) and the fitting equation used is slightly modified according to the expression 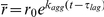. Basic aggregation units are formed over a longer time and their average size is smaller (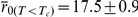) (Tab.1). At times larger than 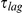 an exponential increase of the hydrodynamic radius, 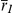, is still observed. In order to verify if aggregations below 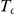, although different with respect to those observed at 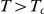, are still consistent with an RLCA process, we investigate the shape of the cluster-number distribution (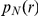) that can be recovered directly from the intensity autocorrelation function [24], [33]. In Fig. 3 we report representative distributions of 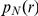 taken at different times during the aggregation occurred at 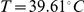. Number weighted radius distributions shift toward higher values with time, and, more relevant, all distributions appear broad and highly asymmetric. Fits of Eq.12 to experimental data evidence that, in agreement with the RLCA aggregation theory, the 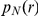 is well described by a power law up to the cut-off radius, 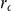, after which it becomes an exponential distribution. Contextually, the fractal dimension does not vary as aggregation proceeds, keeping an average value of 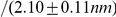. This peculiar behavior imply an universal scaling of the number distribution, i.e. 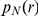 depends only on the cut-off radius 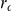 that increases exponentially with time as 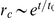 (see inset of Fig. 3) [30], while is independent on the detailed nature of the aggregating particle. In Fig. 4 we report the whole aggregate's size distribution, recovered from aggregations occurred at two different temperatures, below (full dots) and above 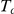 (open diamonds), when the average radius reach a specific value (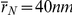). The overlap of the aggregates' size distribution curves before and after the structural transition is a further evidence that the shape of aggregates' size distribution is independent on the size of the aggregating particle (which has different dimension above and below 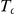) as expected for RLCA aggregations [30].

Therefore above and below 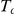 the aggregations are well characterized in the framework of RLCA theory: the final morphology of the aggregates is similar, but the aggregation kinetics are completely different.

**Figure 5:**
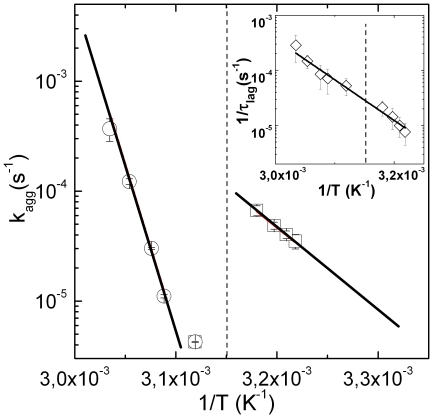
Logarithm of 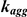 against inverse temperature. Two different behaviors can be identified above and below 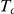 (1/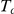 value is indicated by a dashed line). Fit of eq.10 to experimental data allows to determine two different values of the barrier energy: 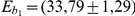
Kcal/mol and 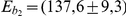 Kcal/mol for the two kinds of aggregation process (below and above 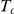 respectively). The rate of formation 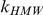 of HMWs, estimated measuring 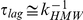, reveal a normal Arrhenius behavior with temperature (inset), revealing that the same abrupt change doesn't involve the first aggregation step.

A closer look of Tab.1, indeed, evidences that the aggregation rate 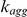 depends on 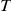 both above and below 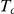, with an abrupt change across 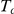. This jump of the 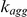 value is a consequence of the phase transition [35] and it can be easily visualized in Fig. 5 where the logarithm of 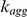 against the inverse temperature is reported. In this plot two different behaviors of 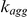 can be identified above and below 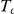 (the 1/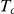 value is indicated by a dashed line). Fit of eq.10 to experimental data allows to recover two distinct energy barriers for the aggregation process: 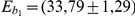
Kcal/mol and 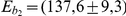 Kcal/mol (below and above 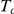 respectively). The rate of formation of HMWs (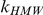), estimated as 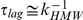 [36], reveals, instead, a single Arrhenius behavior along all the temperature range investigated (inset Fig. 5) showing that the rate of formation of intermediate aggregation units is independent on the alpha-crystallin phase transition.

## Discussion


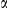
-crystallin is the most abundant lens protein of the mammalian eye, and its aggregates are the main scattering elements strongly involved in the process of cataractogenesis. The increase in light scattering in old and cataractous lenses can be ascribed to alterations in lens crystallins interactions due to age related post-translational modification of 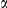-crystallin [5]–[8]. The alterations are triggered by lens cells exposition to elevated temperatures or other stress factors like 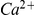 ions, that disrupts the liquid-like molecular order and promote the formation of large scattering particles [9], [10].

Supramolecular structure of crystallins substantially varies both in lenses of different vertebrate species and in various parts of the same lens [37], therefore the understanding of the aggregation mechanism and the fractal clustering could be an important tool to characterize lens ageing and crystallin function.

Here we monitor changes in the 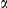-crystallin's aggregation process induced by the thermal structural transition (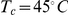).

At all the temperatures investigated supramolecular aggregation of 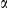-crystallin could be described according to the reaction limited cluster-cluster aggregation theory. Aggregation of the 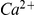 and heat-modified proteins occurs initially by rapidly forming the first clusters or basic aggregating units, corresponding to HMW [20], [38]. After that clusters themselves continue to diffuse, collide, and aggregate. As the aggregation proceeds, clusters with different masses are formed and stick each other. The average cluster size of aggregating HMWs increases exponentially in time and their fractal dimension 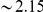 indicates that aggregates sample all the possible mutual configurations before they stick together.

The radius of the HMW is 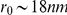, and 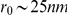, below and above 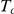, respectively. This difference highlights a substantial alteration in the packing of 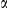-crystallin subunits. Accordingly, HMW molecular weight increases 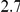 times and HMW concentration decreases by the same factor. A lower HMW concentration reduces the probability of collision decreasing the rate of formation of aggregates. Nevertheless, modifications in subunit's packaging do not affects the kinetic of formation of the HMWs (see inset of Fig. 5).

The aggregation rate, instead, undergoes to an overall abrupt change when crossing 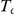. Above 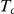, the energy barrier that must be crossed to create larger particles is 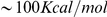 higher than below 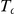. Accordingly, the probability that an activated state occurs along the aggregation process results 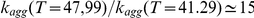 times lower than above 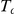. Therefore, the formation of large scattering particles in old and cataractous lenses is inhibited at high temperature by the structural transition that occurs at 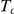. As already pointed out, there is an effective inhibition only of the aggregation step: the rate of aggregation of HMW changes steeply at 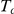, whereas the rate of formation of HMW is unmodified at 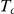.

Therefore, the change in tertiary structure occurring at the endothermic phase transition at 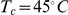 [16], [17], triggers a major reorganization of 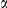-crystallin subunits in the HMWs. A smaller number of more stable HMW particlesis formed, and accordingly the aggregation is inhibited.

Lens crystallin is particularly recessive to deleterious effects from elecromagnetic radiations that are known to be a potential risk factor for cataract and other eyes diseases. Indeed, its aqueous content favors radiation absorption and the very weak vascularization makes difficult to stand fast temperature increases [39].

In this context, the natural self-protective mechanism that we report preserves the lens from premature opacification throughout the lifespan of the organism [40], abruptly reducing the formation of aggregates in the lens fiber cells under hyperthermic conditions, such as those determined by extended exposure to microwaves or other electromagnetic radiation emitted by cell and cordless phones, wireless communications, monitors and even high voltage lines [41]–[45].
